# Giant and Tunable Out-of-Plane Spin Polarization of Topological Antimonene

**DOI:** 10.1021/acs.nanolett.3c00153

**Published:** 2023-07-17

**Authors:** Polina M. Sheverdyaeva, Conor Hogan, Gustav Bihlmayer, Jun Fujii, Ivana Vobornik, Matteo Jugovac, Asish K. Kundu, Sandra Gardonio, Zipporah Rini Benher, Giovanni Di Santo, Sara Gonzalez, Luca Petaccia, Carlo Carbone, Paolo Moras

**Affiliations:** † Istituto di Struttura della Materia-CNR (ISM-CNR), Strada Statale 14 km 163.5, 34149 Trieste, Italy; ‡ Istituto di Struttura della Materia-CNR (ISM-CNR), Via del Fosso del Cavaliere 100, 00133 Roma, Italy; § Dipartimento di Fisica, Università di Roma “Tor Vergata”, Via della Ricerca Scientifica 1, 00133 Roma, Italy; ∥ Peter Grünberg Institut and Institute for Advanced Simulation, Forschungszentrum Jülich and JARA, D-52425 Jülich, Germany; ⊥ Istituto Officina dei Materiali (IOM)-CNR, Laboratorio TASC, Strada Statale 14 km 163.5, 34149 Trieste, Italy; # Peter Grünberg Institut PGI, Forschungszentrum Jülich, 52425 Jülich, Germany; ∇ International Center for Theoretical Physics (ICTP), Trieste, 34151, Italy; • Materials Research Laboratory, University of Nova Gorica, Vipavska 11c, Ajdovščina 5270, Slovenia; ° Elettra - Sincrotrone Trieste S.C.p.A., Strada Statale 14 km 163.5, 34149 Trieste, Italy

**Keywords:** density functional theory, spin-resolved ARPES, electronic structure, topological insulators, 2D materials, antimonene

## Abstract

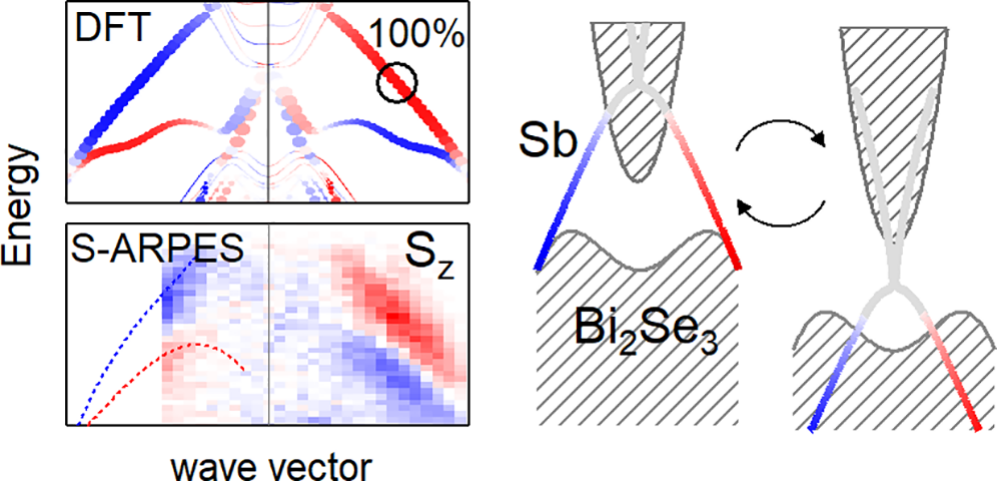

Topological insulators are bulk insulators with metallic and fully spin-polarized surface states displaying Dirac-like band dispersion. Due to spin-momentum locking, these topological surface states (TSSs) have a predominant in-plane spin polarization in the bulk fundamental gap. Here, we show by spin-resolved photoemission spectroscopy that the TSS of a topological insulator interfaced with an antimonene bilayer exhibits nearly full out-of-plane spin polarization within the substrate gap. We connect this phenomenon to a symmetry-protected band crossing of the spin-polarized surface states. The nearly full out-of-plane spin polarization of the TSS occurs along a continuous path in the energy–momentum space, and the spin polarization within the gap can be reversibly tuned from nearly full out-of-plane to nearly full in-plane by electron doping. These findings pave the way to advanced spintronics applications that exploit the giant out-of-plane spin polarization of TSSs.

Topological insulators (TIs) are a class of materials with high spin–orbit coupling (SOC) and nontrivial band topology.^1^ They are insulating in the bulk and metallic at the surfaces due to the presence of robust Dirac-like topological surface states (TSSs) protected by time-reversal symmetry. TSSs are fully spin-polarized with a predominantly in-plane spin orientation near the Fermi level (*E*_F_) as dictated by the symmetry of the spin-momentum locking. The charge–spin interconversion effects arising from this spin texture have remarkable applications in spintronics.^2^ For example, the injection of an electric current into a TI is known to generate in-plane spin-transfer torques (STTs) orders of magnitude larger than in other materials with high SOC.^3^ These giant STTs are suitable for low-power current-induced magnetization switching in TI/ferromagnetic layer heterostructures.^3^

A new spectrum of spintronic effects and related applications would open up if TIs with out-of-plane surface spin texture could be designed.^4,5^ In the context of current-induced magnetization switching, out-of-plane STTs are expected to be more efficient than in-plane STTs in reversing the magnetization of layers with perpendicular magnetic anisotropy, which are typically used in logic and memory devices.^6^ Nonetheless, to date, only a few TSSs have shown significant out-of-plane spin polarization. Record values above 20% have been experimentally reported for Bi_2_Te_3_,^7^ Sb_2_Te_3_,^8^ PbBi_2_Te_4_,^9^ and BiTeI,^10^ yet they remain much lower than the corresponding in-plane spin components. For this reason, phenomena associated with the out-of-plane spin polarization of the TSSs are still poorly explored experimentally.^4,11^

The spin texture of a prototypical TI, Bi_2_Se_3_, was reported to be strongly modified upon the formation of an interface with a bilayer (BL) of honeycomb-like (β-) antimonene.^12^ The topological proximity effect leads to a hybrid TSS that is confined within the antimonene BL and the topmost Se layer (hereon, TSS_Sb_). A reversal of the in-plane spin texture and a significant out-of-plane spin polarization were noted in density functional theory (DFT) calculations.^12^ Here, we provide for the first time a clear experimental demonstration of the phenomenon by means of spin- and angle-resolved photoemission spectroscopy (spin-ARPES) and provide a quantitative, microscopic analysis of its origin using DFT. We show that the out-of-plane spin polarization of TSS_Sb_ reaches giant values of up to 94% within the substrate gap. We connect its emergence to a protected band crossing of fully polarized surface states along . Similar band crossings of surface states were recently reported as two-dimensional analogs of tilted Weyl cones.^13,14^ Importantly, the nearly full out-of-plane spin polarization of TSS_Sb_ is not limited to a certain direction but occurs among a wide range of wave vectors. We demonstrate that the degree of out-of-plane spin polarization of TSS_Sb_ inside the Bi_2_Se_3_ gap can be finely and reversibly tuned by the adsorption of alkali metals or carbon oxide. We propose that similar spin textures may occur in other systems of heterostructures comprising TIs and that such interfaces can be seen as a viable way to tailor the out-of-plane spin texture of TIs.

We recall the electronic structure of the heterostructure formed by one BL of antimonene (i.e., one layer of strongly buckled honeycomb-like β-antimonene^15^) and the Bi_2_Se_3_ substrate.^12,16,17^Figure 1(a) shows the calculated band structure along the  direction (*k*_*x*_ axis). The color scale indicates the spatial localization of the electronic states. Three bands are located within the fundamental gap of the substrate (gray dashed lines indicate the gap edges):^18^ P is localized in antimonene, TSS_Sb_ in antimonene and the topmost Se layer (Figure 1(e), by close analogy to the case of Bi-terminated Bi_2_Te_3_^19^), and B at the interface. TSS_Sb_ can be seen as the migration of the TSS of the substrate (TSS_BS_) to the antimonene BL upon formation of the heterostructure (topologization of antimonene by the proximity effect).^12,16,20^ The Dirac point D lies 0.2 eV above *E*_F_ in a local gap of the Bi_2_Se_3_ conduction band. The three aforementioned bands can be identified in the ARPES spectra of Figure 1(b) (*h*υ = 14 eV). At this photon energy, the Bi_2_Se_3_-related spectral features are strongly suppressed, thus allowing for better visibility of antimonene-derived states. (See for comparison ref (12) and Figure S1(a).) TSS_Sb_ and B (green and light-blue dashed lines) are in good agreement with the DFT calculations (see also Figure S2(a,b) and ref (12)), while P (yellow dashed line) lies at higher binding energies than expected (Figure S2(c,d)). The gray dashed lines indicate the experimental position of the Bi_2_Se_3_ fundamental gap in the antimonene/Bi_2_Se_3_ heterostructure (Figure S1(a)). TSS_Sb_ crosses the entire gap, and only the top part of B enters the gap. P lies below the bottom of the gap, where it overlaps and hybridizes with the Bi_2_Se_3_ states. Thus, the electronic structure and spin structure of the system within the fundamental gap of Bi_2_Se_3_ are determined by TSS_Sb_ and B, on which we focus from here onward.

**Figure 1:**
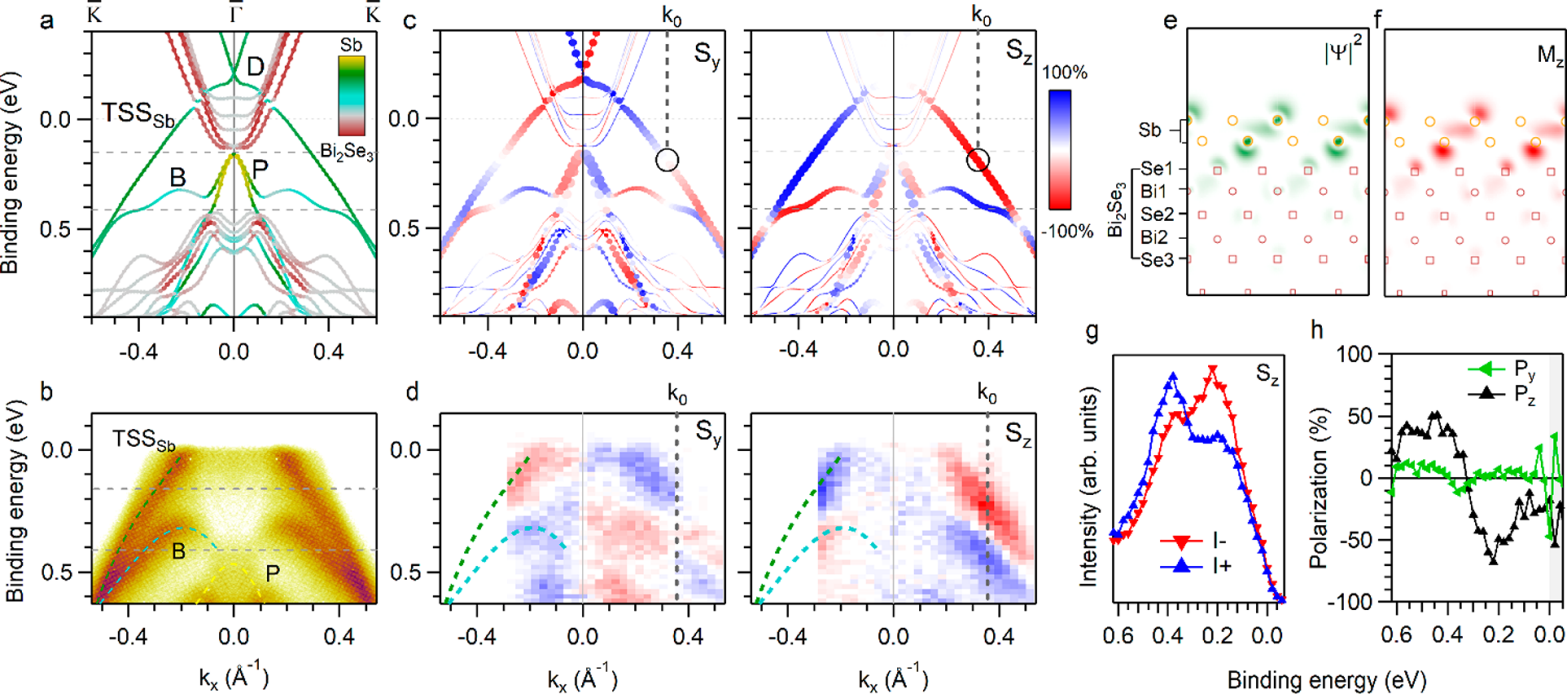
Theoretical and experimental electronic structure of antimonene/Bi_2_Se_3_ along the  direction. (a) Band structure calculations. The color scale indicates the localization of the states. (b) ARPES data taken at *h*ν = 14 eV. (c) Calculated in-plane (left) and out-of-plane (right) spin components. The size of the data points indicates the projection onto the Sb atoms. (d) Experimental in-plane (left) and out-of-plane (right) spin- and angle-resolved photoemission intensities measured with *h*ν = 16 eV. (e) Charge density profile and (f) out-of-plane magnetization distribution of TSS_Sb_ (through the plane intersecting the Sb–Sb bond and topmost Se atom) at the position marked by a circle in panel (c). (g) Out-of-plane spin-resolved photoemission spectra taken at *k*_0_. (h) Spin polarization spectra for the in-plane and out-of-plane spin components at *k*_0_.

Figure 1(c) shows the calculated in-plane (S_*y*_) and out-of-plane (S_*z*_) spin components for the electronic states of antimonene/Bi_2_Se_3_. TSS_Sb_ is fully in-plane spin-polarized near D, by close analogy to the behavior of TSS_BS_.^21^ Its S_*y*_ component decreases steeply below D and reverses at *k*_0_ = 0.35 Å^–1^ (black circle at 0.19 eV), in agreement with the literature.^12,16^ We quantitatively analyzed the S_*z*_ component of TSS_Sb_ at *k*_0_ and observed a surprisingly high value of close to 94%. It remains larger than S_*y*_ throughout the fundamental gap of Bi_2_Se_3_ and reaches about 57% at *E*_F_. B shows a similar predominance of S_*z*_ over S_*y*_ and an opposite spin texture with respect to TSS_Sb_.

The spin-ARPES data of Figure 1(d) confirm the predicted properties of TSS_Sb_ and B. The in-plane spin textures of TSS_Sb_ and B are reversed at *k*_0_. At this point, the out-of-plane spin polarization *P*_*z*_ is about 66% for TSS_Sb_ and 48% for B, and the in-plane ones are close to zero (Figure 1(g,h) and Figure S3(a)). These as-measured out-of-plane spin polarizations are about twice as large as the so far experimentally reported values for the TSSs, despite being lower, as typically observed, than theoretically predicted due to extrinsic and intrinsic effects.^7,8,11^ While referring to the total spin polarization ,^8,11^ the *P*_*z*_ values at *k*_0_ reach 99 and 98% for TSS_Sb_ and B, respectively, in very good agreement with the values predicted by DFT. The complete inversion of S_*y*_ and S_*z*_ textures at opposite *k*_*x*_ values (Figure 1(d)) and the agreement with the DFT calculations show that the present measurements capture the spin polarization of TSS_Sb_ and B in the initial state,^11,22^ which is the relevant property for spintronics. As expected from symmetry considerations, *P*_*z*_ is zero along  (Figure S3(b)).

The spin texture of TSS_Sb_ within the Bi_2_Se_3_ fundamental gap differs remarkably from that of other TSSs in the absolute size of the (giant) S_*z*_ values and in the predominance of S_*z*_ over S_*y*_. For example, the predicted and measured S_*z*_ values for the TSSs of Bi_2_Te_3_^7,8^ and PbBi_2_Te_4_^9^ are 30–40% and about 20%, respectively. In Bi_2_Te_3_, the out-of-plane spin component is very small at *E*_F_, increases moving away from the Dirac point, and reaches its maximum above *E*_F_.^7^ In the case of antimonene/Bi_2_Se_3_, instead, S_*z*_ of TSS_Sb_ is giant within the substrate’s gap and remains predominant between *E*_F_ and 0.5 eV. So far, giant S_*z*_ values have been reported only for the states of transition-metal dichalcogenides^23,24^ or Bi films,^25^ which lack topological protection.^26^ Another important difference with respect to these materials concerns the spatial properties of TSS_Sb_ where S_*z*_ is maximal (circle in Figure 1(c)). Here, TSS_Sb_ displays a high surface localization (Figure 1(e)) and a uniform orientation of the magnetization pattern (Figure 1(f)), whereas the magnetization pattern in WSe_2_ and Bi_2_Te_3_ switches from positive to negative moving from one atomic plane to the next.^7,24,27^ This high surface spin localization and absence of the spin reversal may lead to large spin decoherence times, which are appealing for spintronic applications.^7,28^

In order to explore the origin of the unusually high S_*z*_ values of TSS_Sb_ and B, we followed the evolution of their spin texture by artificially increasing the distance between antimonene and Bi_2_Se_3_ (Figure S4). The calculated S_*z*_ is weak in both nearly free-standing antimonene and Bi_2_Se_3_; therefore, the observed giant S_*z*_ is an exclusive property of the heterostructure. In contrast to WSe_2_, where states with dominant d_*z*_^2^/p_*z*_ character exhibit strongly suppressed S_*z*_,^24^ the charge density profile has a dominant p_*z*_ component^16^ that hardly changes as a function of distance (Figure S5). According to ref (29), the presence of an in-plane electric field can also give rise to a high S_*z*_. We analyzed different stacking arrangements of antimonene and Bi_2_Se_3_ atomic layers (Figure S6) and found that the out-of-plane spin texture is reversed only when the *substrate* stacking—and thus the local potential gradient—is modified. However, since the out-of-plane electric field is normally much larger than the in-plane one, these results would not explain the unusually high, almost fully out-of-plane spin polarization.

In Figure 2, we demonstrate that the giant S_*z*_ values of TSS_Sb_ and B are derived from the constraint imposed by mirror symmetry on their in-plane spin texture. Figure 2(a) shows the band structure of the heterostructure along the  direction. We can observe TSS_Sb_ and B bands that cross close to the 0.25 eV binding energy. The top (bottom) row of Figure 2(b) shows the in-plane (out-of-plane) spin components of TSS_Sb_ and B along radial axes comprised between  and . Since the spin projection along the wave vector direction is forbidden by the symmetry of the Rashba model,^30^ the sum of the spin components shown in the top and bottom rows is always 100% for every state. In particular, along  both states are fully in-plane spin-polarized. Similar band crossings of fully polarized surface states were recently reported experimentally and referred to as two-dimensional analogs of tilted Weyl cones.^13,14^ The mirror symmetry along  protects their accidental crossing from opening a gap. Moving away from  toward , the symmetry constraint vanishes and a gap can open (Figure S7). Since the spin texture of the bands far from the gap region is maintained, the in-plane spin component is forced to become zero while approaching the gap (top row). This results in the emergence of nearly full out-of-plane spin components with opposite signs for TSS_Sb_ and B (bottom row). We emphasize that TSS_Sb_ and B display giant S_*z*_ not only along  but everywhere in the momentum space, except along the six  axes. This is qualitatively different from the S_*z*_ of TSSs of TIs related to their hexagonal warping, that reaches its maximum along . The points where S_*z*_ is maximal, i.e., close to 100% (two gray circles mark these points in the central panels of Figure 2(b), as an example), form the two wavy contours displayed in Figure 2(c). Importantly, they are fully located within the bulk band gap of the substrate.

**Figure 2:**
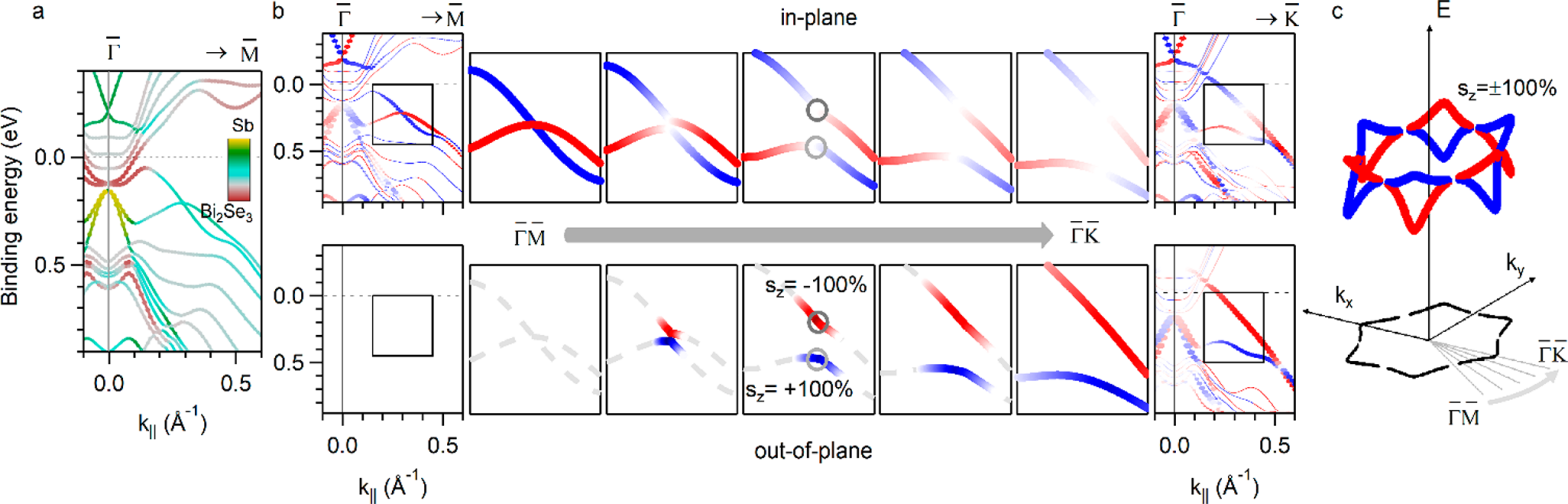
Origin of the giant out-of-plane spin texture. (a) Band structure calculations along . The color scale indicates the localization of the states. (b) Spin-resolved band structure. Top row: in-plane spin component of the TSS_Sb_ and B bands along radial axes comprised between  and . Bottom row: same as the top row for the out-of-plane spin component. Along , the computed S_*z*_ is zero. Gray circles mark the states with zero in-plane and ±100% out-of-plane spin components in the central panels. Band dispersions away from high-symmetry directions are shown schematically. (c) Schematic representation of the S_*z*_ = ±100% contours for TSS_Sb_ and B in the energy–momentum space.

We now explore the possibility of tuning the out-of-plane spin texture of antimonene/Bi_2_Se_3_ via electron doping. Figure 3(a) shows the band structure of the system with a 0.25 monolayer (ML) of K on top of antimonene in the most stable atomic configuration. (Section 2 in the Supporting Information, Figures S8–S10, and Table S1 consider other structures and coverages.) Here, 1 ML corresponds to the density of the uppermost Sb plane. The K-induced electron doping influences the antimonene- and Bi_2_Se_3_-related features differently due to the presence of a surface dipole (Figure S11). D is shifted toward higher binding energy by 0.7 eV (green arrow at 0.5 eV below *E*_F_) and toward the bulk Bi_2_Se_3_ bands by only 0.3 eV. This relative energy shift strongly alters the spin texture of the system (Figure 3(b)). Now TSS_Sb_ shows pronounced S_*y*_ and negligible S_*z*_ components within the entire fundamental gap of Bi_2_Se_3_. (B becomes degenerate with the bulk valence states of the Bi_2_Se_3_.) This behavior is expected on the basis of the spin structure of TSS_Sb_ above D (Figure 1(c)) and the analogous relative shift between TSS_BS_ and the bulk bands observed upon alkali metal deposition on Bi_2_Se_3_.^31−33^ Notably, TSS_Sb_ retains the strongly localized character (Figure 3(c)) and the constant out-of-plane magnetization pattern (Figure 3(d)) of the pristine antimonene/Bi_2_Se_3_ system.

**Figure 3:**
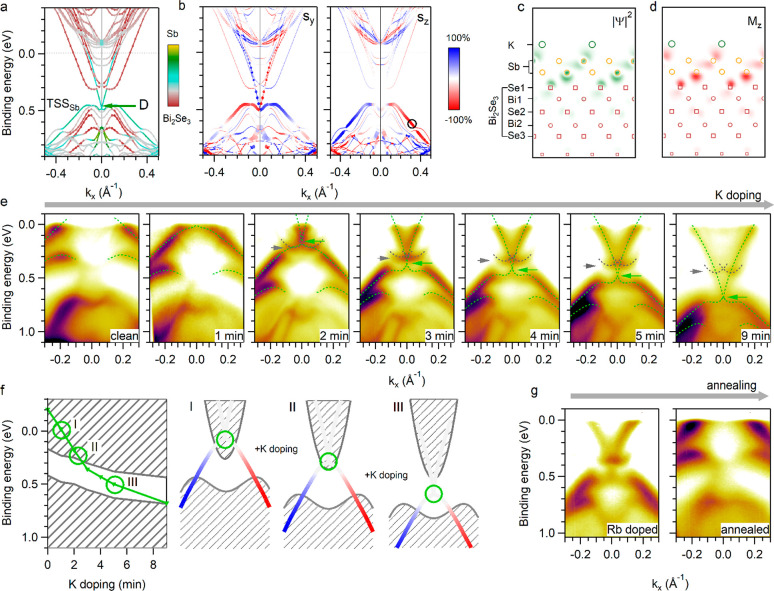
Tailoring of the spin texture by electron doping. (a) Band structure calculations for 0.25 ML K/antimonene/Bi_2_Se_3_. The color scale indicates the localization of the states. (b) Calculated in-plane (left) and out-of-plane (right) spin components. The size of the data points indicates the projection onto the Sb atoms. (c) Charge density profile and (d) out-of-plane magnetization distribution of TSS_Sb_ at the position marked by a circle in panel (b). (e) ARPES spectra along  as a function of the K coverage, *h*υ = 14 eV. (f) Position of D (green) with respect to the bulk band edges (gray) and the schematics of the out-of-plane spin texture of TSS_Sb_ as a function of the K deposition. (g) ARPES spectra of antimonene/Bi_2_Se_3_ doped by Rb before and after annealing to 350 K.

The electronic structure shown in Figure 3(a) is actually observed by ARPES when K is deposited on the surface of antimonene/Bi_2_Se_3_ (Figure 3(e)). As the amount of K increases, TSS_Sb_ and B (green dashed lines) continuously shift toward higher binding energies. D (green arrow) becomes visible from 2 min deposition and reaches 0.7 eV at saturation coverage. Starting from the 2 min deposition, we also observe two inverted parabolas (gray dashed lines) that shift toward higher binding energy as the K doping increases (Figure S1(b–d)). By comparison with the calculations of Figure 3(a) and the ARPES data of K-doped Bi_2_Se_3_,^31^ these bands can be identified with the bottom of the bulk conduction band of Bi_2_Se_3_. Since the size of the fundamental gap of Bi_2_Se_3_ is known (0.25 eV), it is possible to locate D with respect to the bulk band edges at each K deposition step, as shown on the left side of Figure 3(f). The resulting electronic structure of the system, regarding TSS_Sb_ only, is schematically shown on the right side of Figure 3(f). In pristine or lightly K-doped antimonene/Bi_2_Se_3_ (I), the spin texture in the fundamental gap of Bi_2_Se_3_ is dominated by states with giant S_*z*_. With increasing K coverage, the S_*z*_ component of TSS_Sb_ decreases (II) and eventually disappears (III).

A similar electron doping effect can be induced by dosing Rb or CO on antimonene/Bi_2_Se_3_ held at liquid nitrogen temperature (left side of Figure 3(g) and Figure S12). Also in this case, we observe the downward shift of TSS_Sb_ and B and the appearance of two inverted parabolas, by close analogy to the K-doped system. Similar to Bi_2_Se_3_,^31^ the effect of K, Rb, and CO dosing can be removed by raising the temperature of the antimonene/Bi_2_Se_3_ system, thus making the electron doping effect largely reversible (right side of Figure 3(g) and Figure S12) and repeatable (Figure S13).

Our analysis demonstrates that it is possible to design topological systems with giant S_*z*_ components by interfacing existing TIs with trivial materials, such as semiconducting antimonene. The antimonene/Bi_2_Se_3_ heterostructure features a single spin channel inside the bulk gap with a giant out-of-plane spin polarization and topological protection, which is strongly localized in the surface. This system satisfies an important requirement for spintronic applications, i.e., the tunability of spin- and electronic properties.^34−37^ The out-of-plane spin polarization can indeed be finely and reversibly tuned from almost 100% to 0%. This final state can also be reached via absorption of other elements, such as Mg and Ca.^38^ The simple band structure described above can be used as a model for the simulation and experimental testing of transport properties for spintronic devices and could be implemented in magnetic memory applications.

The mechanism described above, in which a fully out-of-plane spin polarization arises from a symmetry-protected Weyl-like band crossing, to the best of our knowledge has not been reported in the literature. We suggest that it may manifest in other systems that feature protected band crossing along a line of mirror symmetry. In the context of topologically protected states, a band crossing along the  direction and exhibiting a similar in-plane spin texture reversal was observed in the related system of double BL antimonene on Bi_2_Se_3_.^12^ A protected band crossing followed by a nearly full S_*z*_ may well exist also in similar heterostructures that manifest a topological proximity effect, i.e., antimonene on Bi_2_Te_3_, Sb_2_Te_3_, and Bi_2_Se_2_Te.^16,39,40^ A crossing of spin-polarized surface bands along a high-symmetry line was experimentally reported for NbGeSb^13^ and W(110).^14^ Although these states lack topological protection, one may also expect full out-of-plane spin polarization away from mirror symmetry planes in these systems.

## Methods

High-quality single crystals of Bi_2_Se_3_ were grown by the Bridgman method. Bismuth and selenium were purchased from Sigma-Aldrich at 99.999% and ≥99.5% trace metal basis, respectively. The stoichiometric amounts of high-purity elements were sealed in evacuated quartz ampules and heated to 1023 at 21 K/h. The ampules were maintained at that temperature for 48 h. Thereafter, the temperature was slowly reduced to 523 K at 5 K/h and then cooled to room temperature.

Sb was deposited on freshly cleaved Bi_2_Se_3_ at room temperature and subsequently annealed to 430 K to form the β-antimonene phase as described in refs (12), (17), and (41). Potassium and rubidium were deposited on the sample kept at 80 K at a rate of 0.04 monolayer (ML)/min (as referenced to the density of the Bi_2_Se_3_ surface). CO adsorption was achieved by cooling the sample from room temperature to 80 K in a CO atmosphere. The LEED pattern showed sharp 1 × 1 spots at all preparation stages (Figure S14).

DFT calculations were performed within a plane-wave/pseudopotential framework as implemented in the quantum-ESPRESSO (QE) code.^42^ Spin–orbit coupling was incorporated via use of fully relativistic ultrasoft pseudopotentials^43,44^ with a kinetic energy cutoff of 45 Ry. The Bi_2_Se_3_ surface was modeled by using a centrosymmetric slab containing six quintuple layers. The four topmost atomic layers (plus the antimonene layer and the K atoms) were allowed to relax during structural optimization. The experimental lattice constant of 4.143 Å was used.^45^ The PBE functional^46^ and Grimme-D2 van der Waals correction^47^ were adopted. This computational scheme yielded results in close agreement with our previous studies using QE and VASP.^12,17^ The equilibrium distance *d*_0_ = 2.32 Å and the 1 × 1 lattice matching considered in the calculations are justified in previous structural studies of the system.^12,20^ Adsorption of potassium was simulated using 1 × 1, 2 × 2, and 3 × 3 supercells, corresponding to coverages of 1, 0.25, and 0.11 ML. Several on-top and intercalated geometries were investigated, as shown in Section 2 of the Supporting Information.

The ARPES experiments were carried out at the APE-LE,^48^ BaDElPh,^49^ and VUV-photoemission beamlines of the synchrotron Elettra (Trieste, Italy) with *h*υ = 14–60 eV and *p*-polarized light, while keeping the sample at 80 K. The electron spectrometers were placed at an angle between 42° and 50°, with reference to the direction of the incoming *p-*polarized photon beam. The energy resolution was about 20 meV, and the momentum resolution was about 0.02 Å^–1^. Spin-resolved ARPES data were taken at the APE-LE beamline by using a Scienta-Omicron DA30 hemispherical analyzer operating in deflection mode and equipped with two orthogonal VLEED reflectometers, with an energy resolution of about 90 meV and a momentum resolution of about 0.04 Å^–1^. During the spin measurements, the analyzer’s slits were set parallel to the  direction, and the spin-ARPES spectra along high-symmetry directions were obtained by using the deflectors without moving the sample. The spin polarization was calculated as described in ref (48) and using the Sherman function value *S* = 0.26.

## Data Availability

The data that support the findings of this study are available within the article and Supporting Information. Extra data are available from the corresponding authors upon reasonable request.

## Supporting Information Available

Additional experimental data (ARPES and spin-ARPES data, LEED patterns) as well as DFT calculations useful to understanding the theoretical results shown in the main text (PDF)
