# Supplementary material for: Giant and Tunable Out-of-Plane Spin Polarization of Topological Antimonene
Source: Nano Lett. 2023 Jul 17;23(14):6277–83. doi: 10.1021/acs.nanolett.3c00153 (PMC10375579; doi:10.1021/acs.nanolett.3c00153)
Supplement: Supplementary file 1 — nl3c00153_si_001.pdf [file nl3c00153_si_001.pdf]

Supplemental material for

Giant and tunable out-of-plane spin polarization of topological antimonene

Polina M. Sheverdyaeva[1,*¶], Conor Hogan[2,3*¶], Gustav Bihlmayer[4], Jun Fujii[5], Ivana Vobornik[5], Matteo Jugovac[1,6], Asish K. Kundu[1,7], Sandra Gardonio[8], Zipporah Rini Benher[8], Giovanni Di Santo[9], Sara Gonzalez[9], Luca Petaccia[9], Carlo Carbone[1], and Paolo Moras[1]

[1]*Istituto di Struttura della Materia-CNR (ISM-CNR), Strada Statale 14 km 163.5, 34149, Trieste, Italy*

[2]*Istituto di Struttura della Materia-CNR (ISM-CNR), Via del Fosso del Cavaliere 100, 00133 Roma, Italy*

[3]*Dipartimento di Fisica, Università di Roma "Tor Vergata", Via della Ricerca Scientifica 1, 00133 Roma, Italy*

[4]*Peter Grünberg Institut and Institute for Advanced Simulation, Forschungszentrum Jülich and JARA, D-52425 Jülich, Germany*

[5]*Istituto Officina dei Materiali (IOM)-CNR, Laboratorio TASC, Strada Statale 14 km 163.5, 34149, Trieste, Italy*

[6]*Peter Grünberg Institut PGI, Forschungszentrum Jülich, 52425 Jülich, Germany*

[7]*International Center for Theoretical Physics (ICTP), Trieste, 34151, Italy*

[8]*Materials Research Laboratory, University of Nova Gorica, Vipavska 11c, Ajdovščina 5270, Slovenia*

[9]*Elettra - Sincrotrone Trieste S.C.p.A., Strada Statale 14 km 163.5, 34149 Trieste, Italy*

Corresponding Author:

Polina M. Sheverdyaeva, Email: polina.sheverdyaeva@ism.cnr.it

Conor Hogan, Email: conor.hogan@ism.cnr.it

Section 1. Additional ARPES and DFT data

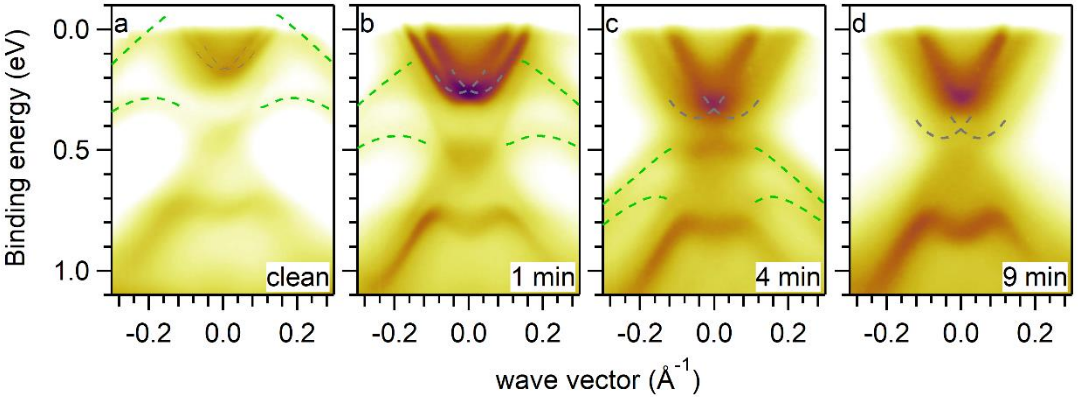

Figure S1. ARPES intensity along $\overline{K\Gamma K}$ direction for antimonene/Bi_2Se_3, (a) clean and (b-d) with different K doping taken with hυ=20 eV. Green dashed lines mark the position of antimonene-derived states. At these experimental conditions the bottom of the conduction band can be identified with the minima of the parabola. The top of the valence band is estimated to be located 0.25 eV below, in agreement with the literature [1].

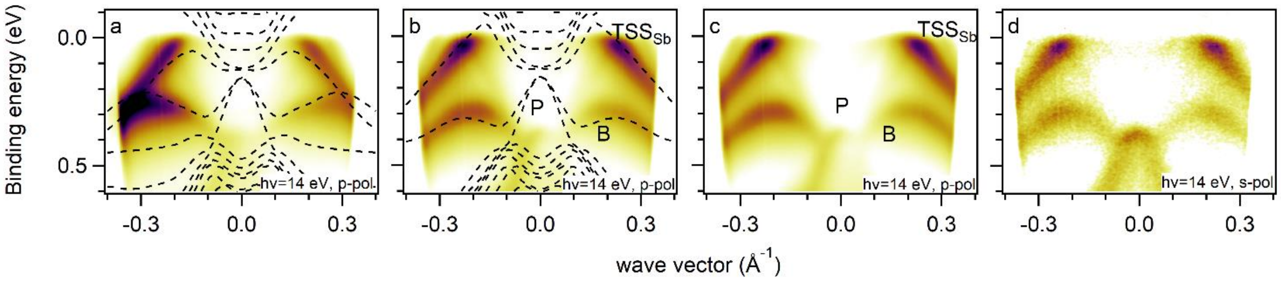

Figure S2. ARPES spectra of antimonene/Bi_2Se_3 for photon energy 14 eV (a,b) overlapped with band structure calculations, *p*-polarized light along: (a) the $\overline{M\Gamma M}$ direction, (b) the $\overline{K\Gamma K}$ direction. (c,d) Polarization dependence of ARPES spectra along $\overline{K\Gamma K}$ taken with: (c) *p*-polarized light (d) *s*-polarized light. The relative intensity of the feature identified with the P band becomes stronger with *s*-polarization, consistent with the purely p_x,p_y character of P [2,3].

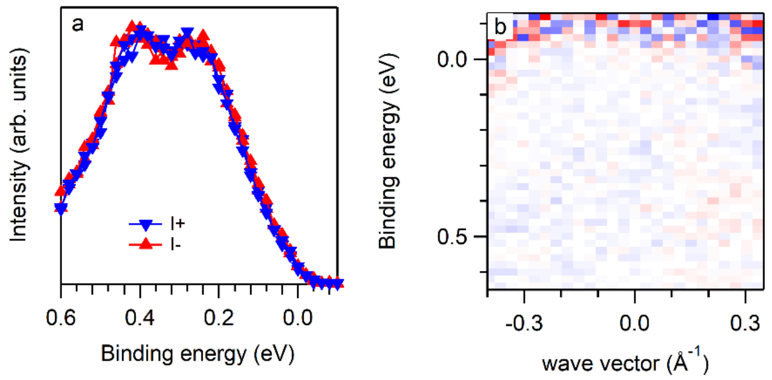

Figure S3. (a) In-plane spin-resolved photoemission spectra taken at k_0 (see Fig. 1 of the main text), hυ=16 eV. The spin-quantization axis is aligned along **y**. (b) Angle-resolved spin polarization taken along the $\overline{M\Gamma M}$ direction, hυ=16 eV. The spin-quantization axis is aligned along **z**.

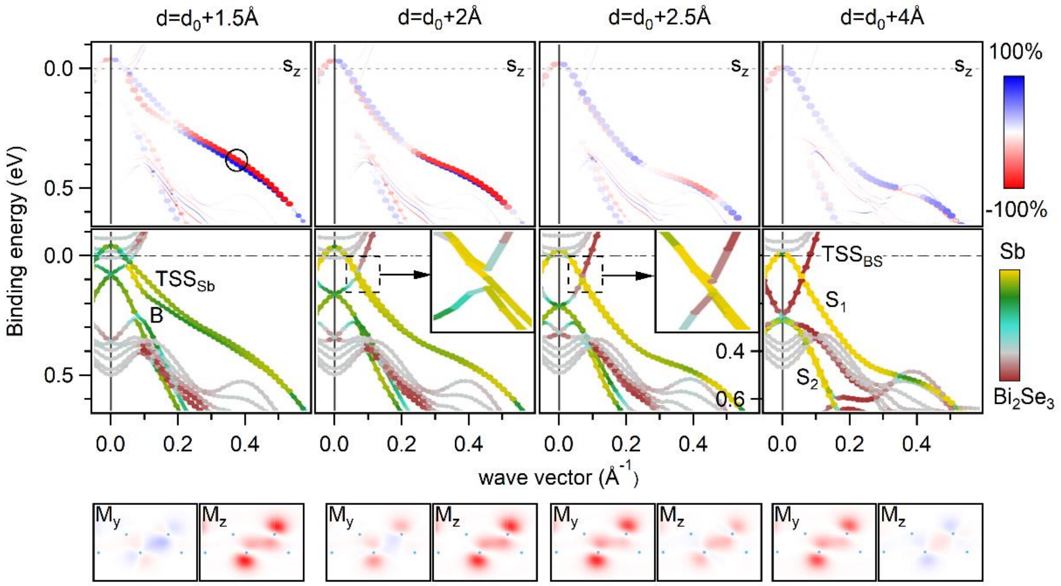

Figure S4. Electronic structure of antimonene as a function of the distance d from the substrate Bi_2Se_3, where d_0 is the equilibrium distance: out-of-plane spin texture (top row), real space projections (middle row) and magnetization for in-plane and out-of-plane directions, zoomed on the antimonene (bottom row). The out-of-plane spin polarization is strongly suppressed when d is increased by more than 2.5Å, as can also be confirmed by the magnetization patterns. At the same distance, the TSS_{Sb} and B band start to merge and become localized fully on antimonene, while the TSS_{BS} is localized fully on Bi_2Se_3.

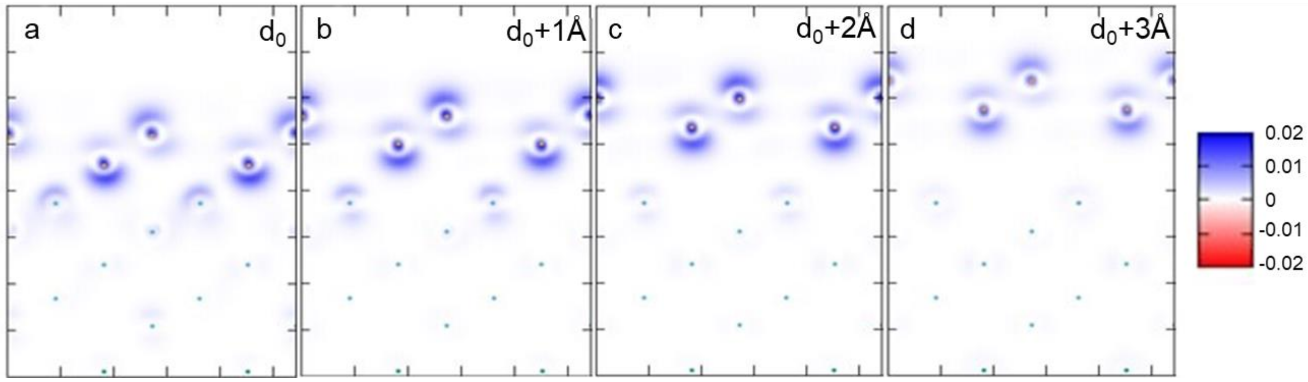

Figure S5. Charge profile of antimonene on Bi_2Se_3 as a function of distance between them (d_0, d_0+1Å, d_0+2Å, d_0+3Å). We observe that the shape of the orbitals hardly changes over the range of distance shown, within which we observed the suppression of the out-of-plane spin polarization.

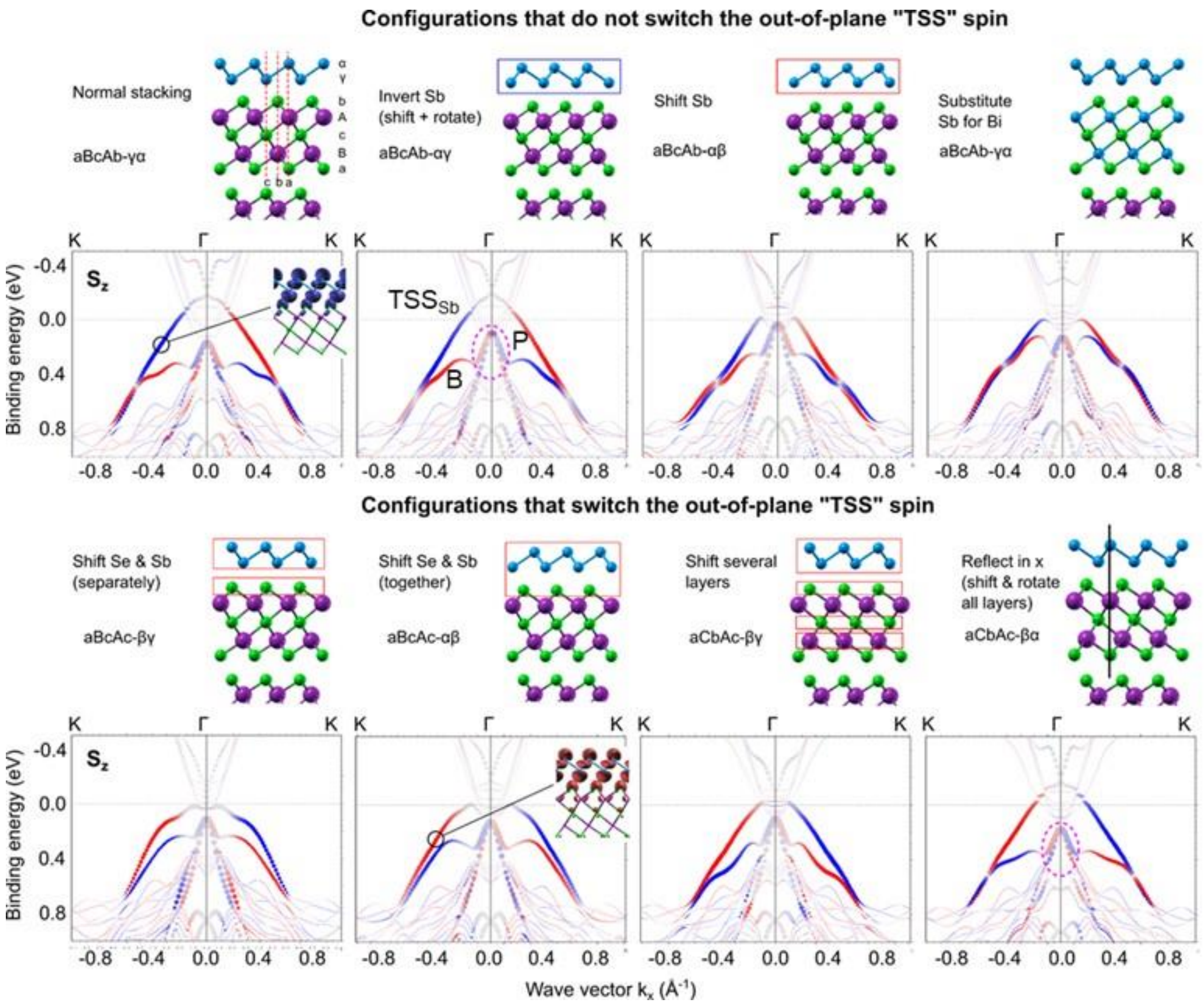

Figure S6. Dependence of out-of-plane spin expectation value on stacking geometry of the Sb atomic layers and Bi_2Se_3 atomic layers in the topmost QL. Se and Bi stacking is labeled with Roman letters; Sb stacking is labeled with Greek letters. The dotted oval highlights spin switching of the Sb-localized P bands with respect to the adjacent panel. Listed changes are reported with respect to the initial (normal stacking) configuration. The insets show the magnetization density at the indicated states: only small differences are noted within the QL. It is clear that switching of S_z of the TSS_{Sb} and B bands only occurs with modification of the *substrate* geometry. This should occur due to a change in the in-plane potential gradient (from hybridization and/or charge redistribution). In contrast, the Sb-localized P bands only change sign with a rotation/inversion of the Sb layer. (Note, not all configurations retain the topological character originating in bulk Bi_2Se_3).

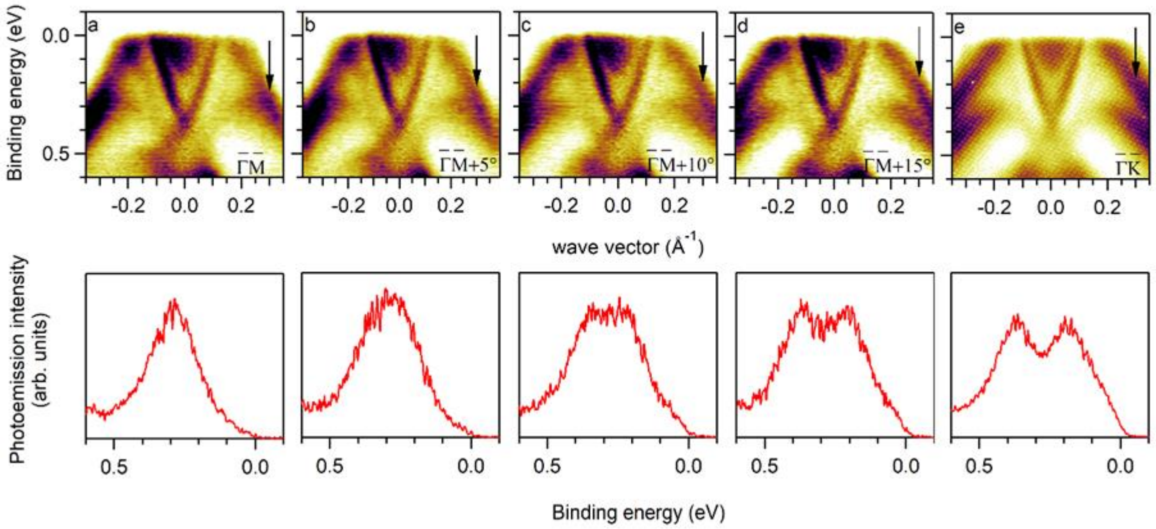

Figure S7. APRES data on an incomplete 1BL antimonene layer (the Bi_2Se_3-derived cone is clearly visible), extracted from 3D volume ARPES data, taken with 16 eV *p*-polarized light. Top row: band structure evolution while moving from $\overline{M\Gamma M}$ towards $\overline{K\Gamma K}$. Bottom row: energy distribution curves extracted at the position of the black arrow. The arrow is fixed at the $k_{||}$ position where a band crossing was observed along $\overline{M\Gamma M}$ direction (panel a).

Section 2. Potassium adsorption on antimonene/Bi_2Se_3

Adsorption of atomic K on clean Bi_2Se_3 and on the antimonene/Bi_2Se_3 hetero-structure was simulated for various coverages using 1×1, 2×2, and 3×3 supercells. The optimal adsorption site was determined using the 1×1 cell by placing the K atom at various high symmetry sites and comparing the relative total energies following structural relaxations. The H3 site was most favoured on clean Bi_2Se_3 and on top of the antimonene layer. For intercalated geometries the H3 site was assumed, and a small selection of antimonene layer positions tested. K adsorption energies were computed with respect to the minimum energy structure for all on-top and intercalated configurations. The most stable geometries are shown in Figure S8.

As reported in the Table 1, intercalated K is energetically more favorable within DFT. However, this configuration is not attainable in our experiments. The adsorbed K was stabilized by maintaining a low temperature throughout the deposition and ARPES measurements.

Computed band structures as a function of K coverage (on-top geometry) are shown in Figure S9. Bands are folded according to the supercell used. Nonetheless, the position of the Dirac point can easily be determined by analysis of the atomic projection and spin expectation values. At 1.0 ML the bands are highly perturbed by atomic orbitals of the K layer.

The band structure for an intercalated geometry at 0.25 ML coverage is instead shown in Fig. S10. It is clear that the bands are almost equivalent to those of a K-doped Bi_2Se_3 slab. The computed bands are in clear disagreement with the experimental data (Fig. 3(e)). For the intercalated geometry no antimonene Dirac cone can be observed, that would contradict our experimental data.

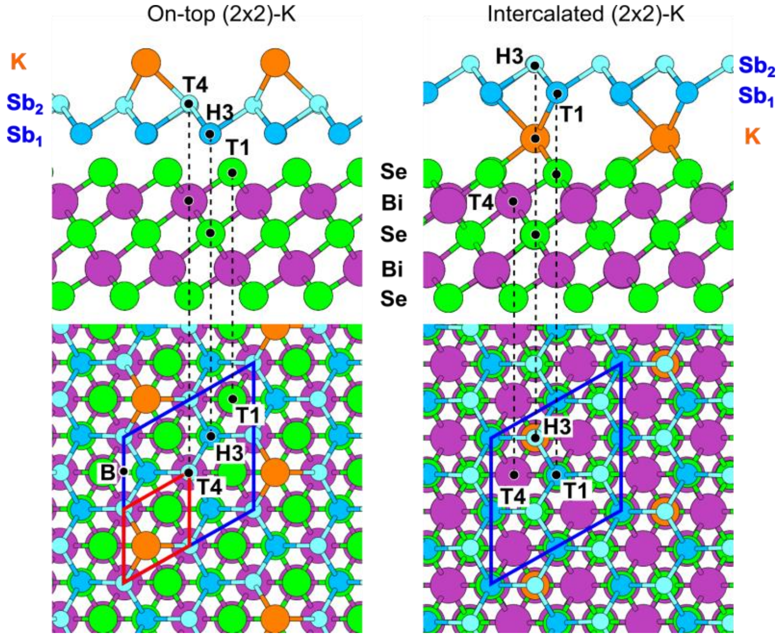

Figure S8. Sb and K adsorption sites on Bi_2Se_3. The blue (red) diamond indicates the 2×2 (1×1) surface unit cell. The most stable on-top (left) and intercalated (right) geometries are shown.

	Sb_1	Sb_2	K	Energy (eV/atom)
1BL	H3	T4	-	
On-top	H3	T4	T1	0.67
	H3	T4	T4	0.99
	**H3**	**T4**	**H3**	**0.66**
	H3	T4	B	0.75
Intercalated	H3	T4	H3	0.30
	T4	T1	H3	0.03
	**T1**	**H3**	**H3**	**0.00**

TABLE 1. Geometries and relative adsorption energies of the K/antimonene/Bi_2Se_3 slabs, assuming a 1.0 ML coverage. Site labels refer to the Bi_2Se_3 surface. The structures indicated in bold are depicted in Figure S8. Energies are given relative to the most stable configuration (last row).

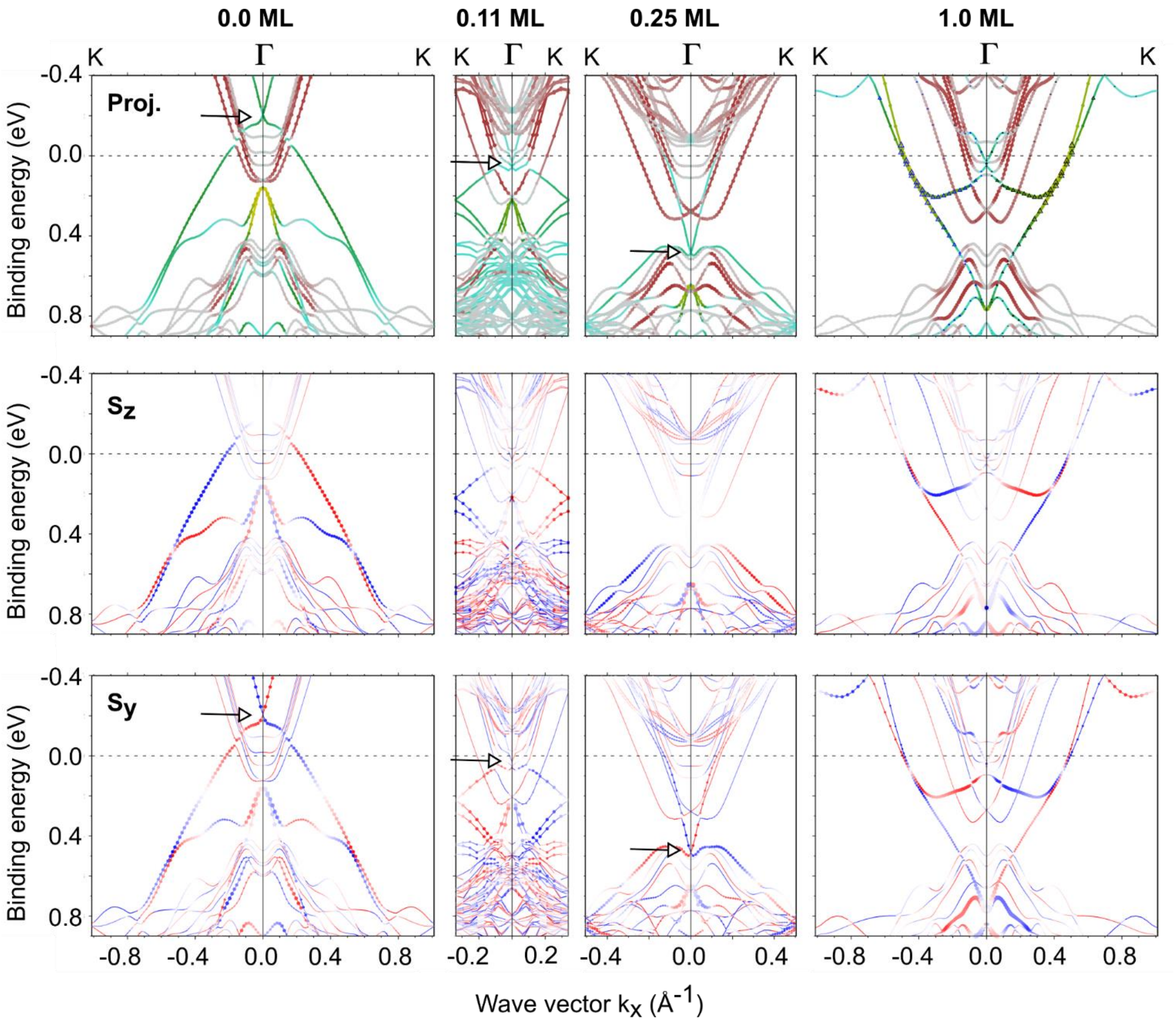

Figure S9. Band structure of the K/antimonene/Bi_2Se_3 hetero-structure as a function of K coverage. The atomic projections (antimonene vs Bi_2Se_3) and spin expectation values (in-plane S_y and out-of-plane S_z) are plotted. Significant projections onto atomic K states are indicated for 1.0 ML with open triangles. Unit cells of size 3×3, 2×2, and 1×1 (left-to-right) containing 1 atom of K are used for defining non-zero coverages. The arrow indicates the position of the Dirac point. Colour schemes as in Fig. 1(a) and 1(c).

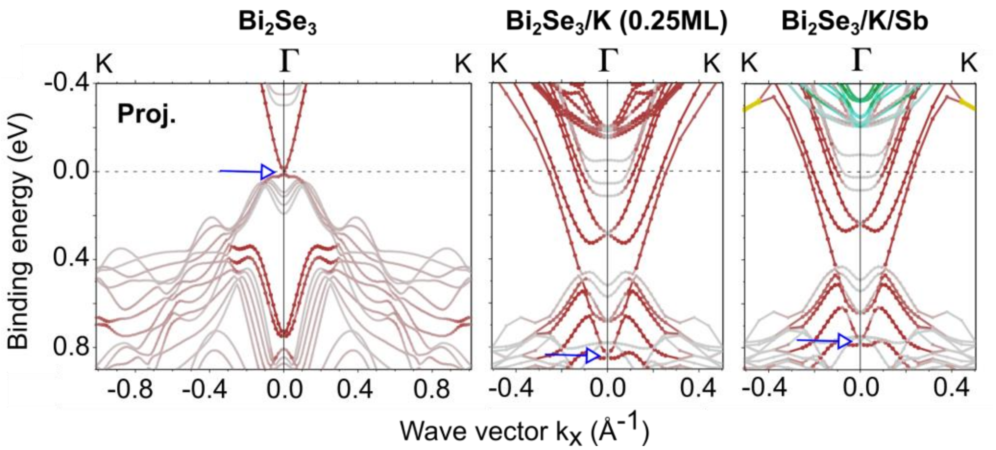

Figure S10. Band structure of clean Bi_2Se_3, K/Bi_2Se_3 and antimonene/K/Bi_2Se_3 (K intercalated). K coverages are 0.25 ML corresponding to a 2×2 surface unit cell. The Dirac point is indicated. Colour schemes as in Fig. 1(a).

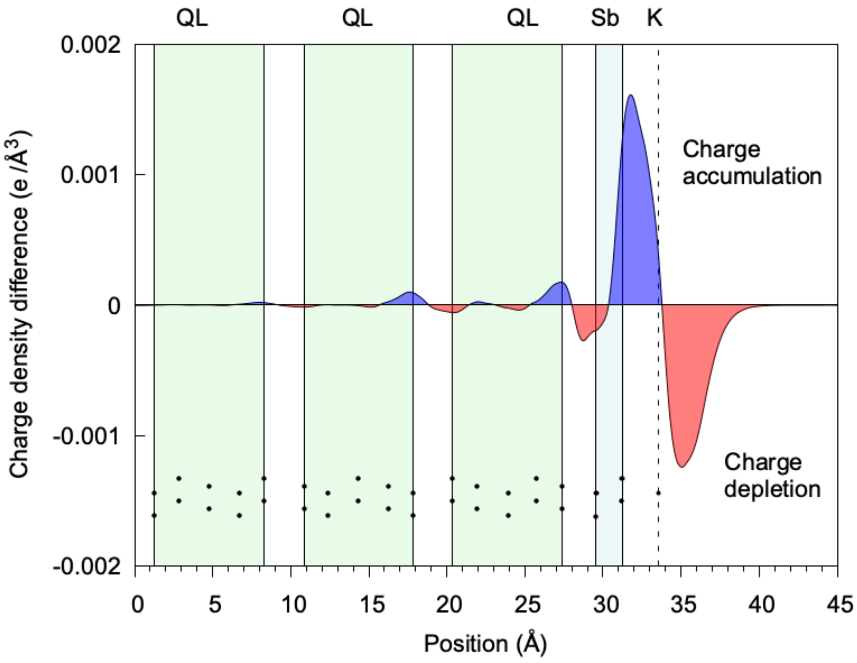

Figure S11. Charge density redistribution following K adsorption on the antimonene/Bi_2Se_3 hetero-structure. K adsorption creates a strong dipole at the antimonene layer and smaller dipoles in the bulk interlayer regions.

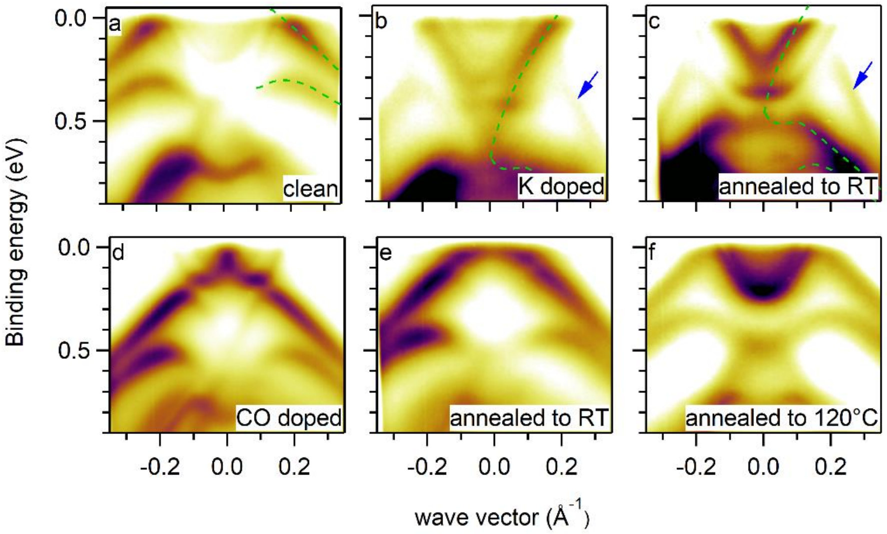

Figure S12. ARPES intensity along the $\overline{K\Gamma K}$ direction for (a-c) ~1.2BL antimonene/Bi_2Se_3 as a function of K adsorption for (a) clean antimonene; (b) with 9 min K on top; (c) same as (b) annealed to room temperature for 10 min. Green dashed lines mark the dispersion of the antimonene-derived states. The blue arrows mark 2BL-derived features (see Ref. [3]) that start to appear at this coverage: they experience a smaller energy shift under K doping with respect to the 1BL-derived ones. (d) with CO dosing for 1BL antimonene/Bi_2Se_3 (e) same as (d) after anneal to room temperature (f) same as (d) after anneal to 120°C for 35 min. Panels (a-e) are taken with hυ=14 eV, panel (f) with hυ=20 eV.

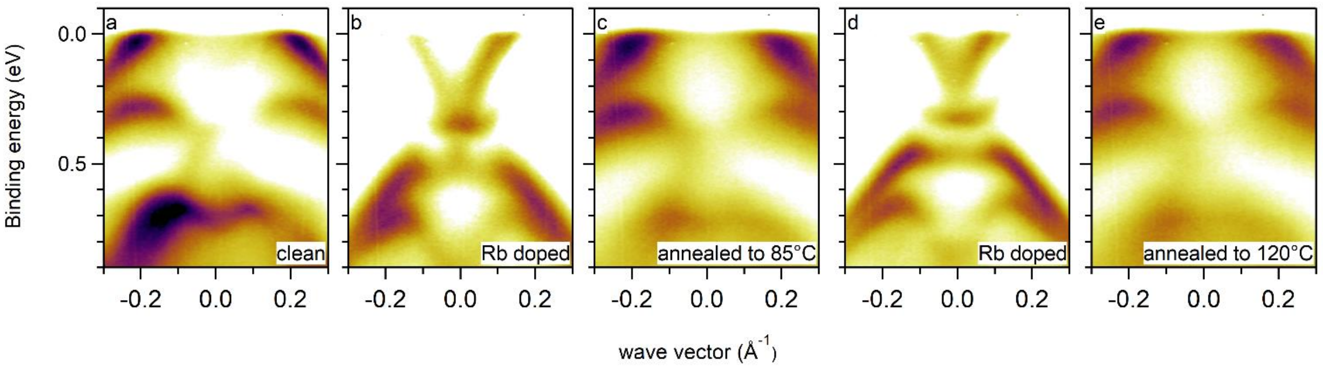

Figure S13. Doping-annealing cycles. ARPES intensity of (a) 1 BL antimonene (b) after deposition of 1 min Rb (c) after annealing to 85°C for 1 h (d) after another 1 min of Rb deposition (e) after annealing to 120°C for 1 h; hv=14 eV.

Section 3. Low energy electron diffraction (LEED)

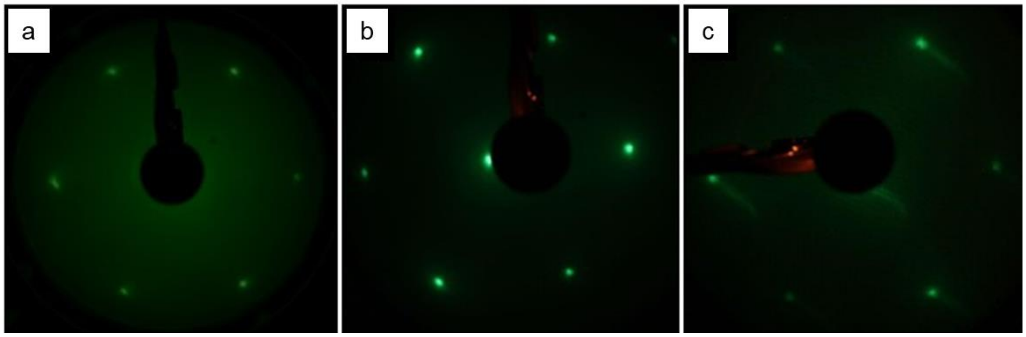

Figure S14. LEED pattern of (a) clean Bi_2Se_3 (E=43.6 eV) (b) antimonene/Bi_2Se_3 (E=62.1 eV), (c) 9 min K/antimonene/Bi_2Se_3 (E=57.7 eV).

References

[1] Nechaev, I. A., et al. Evidence for a direct band gap in the topological insulator Bi_2Se_3 from theory and experiment. *Phys. Rev. B* **2013**, 87, 121111.

[2] Jin, K.-H.., Yeom, H. W., & Jhi, S.-H. Band structure engineering of topological insulator heterojunctions. *Phys. Rev. B* **2016**, *93*, 075308.

[3] Holtgrewe, K. et al. Topologization of β-antimonene on Bi_2Se_3 via proximity effects, *Sci. Rep.* **2020**, 10, 14619.
